# Does the Good Schools Toolkit Reduce Physical, Sexual and Emotional Violence, and Injuries, in Girls and Boys equally? A Cluster-Randomised Controlled Trial

**DOI:** 10.1007/s11121-017-0775-3

**Published:** 2017-04-10

**Authors:** Karen M. Devries, Louise Knight, Elizabeth Allen, Jenny Parkes, Nambusi Kyegombe, Dipak Naker

**Affiliations:** 1 London School of Hygiene and Tropical Medicine, 15-17 Tavistock Place, London, WC1H 9SH UK; 2 University College London-Institute of Education, London, UK; 3 Raising Voices, Kampala, Uganda

**Keywords:** Violence, Bullying, Gender, School-based interventions, Uganda

## Abstract

We aimed to investigate whether the Good School Toolkit reduced emotional violence, severe physical violence, sexual violence and injuries from school staff to students, as well as emotional, physical and sexual violence between peers, in Ugandan primary schools. We performed a two-arm cluster randomised controlled trial with parallel assignment. Forty-two schools in one district were allocated to intervention (*n* = 21) or wait-list control (*n* = 21) arms in 2012. We did cross-sectional baseline and endline surveys in 2012 and 2014, and the Good School Toolkit intervention was implemented for 18 months between surveys. Analyses were by intention to treat and are adjusted for clustering within schools and for baseline school-level proportions of outcomes. The Toolkit was associated with an overall reduction in any form of violence from staff and/or peers in the past week towards both male (aOR = 0.34, 95%CI 0.22–0.53) and female students (aOR = 0.55, 95%CI 0.36–0.84). Injuries as a result of violence from school staff were also lower in male (aOR = 0.36, 95%CI 0.20–0.65) and female students (aOR = 0.51, 95%CI 0.29–0.90). Although the Toolkit seems to be effective at reducing violence in both sexes, there is some suggestion that the Toolkit may have stronger effects in boys than girls. The Toolkit is a promising intervention to reduce a wide range of different forms of violence from school staff and between peers in schools, and should be urgently considered for scale-up. Further research is needed to investigate how the intervention could engage more successfully with girls.

Violence is one of the most pervasive and serious public health problems facing children globally. Physical, sexual and emotional violence, as well as neglect, have a range of short and longer term health consequences, including increased risk of mental disorders (Norman et al. 2012), suicide (Devries et al. 2014b), sexually transmitted infections (Norman et al. 2012) and poor educational attainment (Boden et al. 2007). In most contexts, girls and boys have different risks and experiences of these forms of violence, with boys in some settings being more likely to experience physical violence (Akmatov 2010), and girls being more likely to experience sexual violence (Stoltenborgh et al. 2011). In East Africa, the school environment may be a main location where exposure to various forms of violence occurs, from both school staff and from peers (UNICEF Tanzania, Centers for Disease Control and Prevention, & Muhimbili University of Health and Allied Sciences 2011; United Nations Children’s Fund Kenya Country Office 2012). In Uganda, despite the prohibition of corporal punishment at school, anecdotal evidence indicates that most teachers continue to believe that pain is the only effective motivator for learning. This situation is exacerbated with large classroom sizes and inadequate training on how to engage attention of students. We previously found that more than 90% of students had experienced violence at school and more than 50% had experienced it last week (Devries et al. 2014a).

Relatively few interventions to prevent any form of child maltreatment have been tested in low- and middle-income countries, and fewer still focus on the school environment. The Good School Toolkit, developed by Ugandan NGO Raising Voices, takes a whole school approach to violence prevention and aims to change culture at the school level to promote respect, participation and reflection on concepts and exercise of power. We recently reported that the Good Schools Toolkit can reduce physical violence from school staff to primary school students (Devries et al. 2015). To our knowledge, no other interventions to prevent violence from school staff to students have been rigorously evaluated. We also found evidence of a sex difference in the effect of the Toolkit—although it was effective for both sexes, our analysis suggests (Devries et al. 2015) that it was more effective reducing physical violence from school staff towards boys (OR = 0.34, 95%CI 0.21–0.56), than girls (OR = 0.46, 95%CI 0.29–0.74), *p* value for interaction 0.043.

In this paper, we took a more comprehensive look at the effects of the Good School Toolkit on other forms of violence in the school environment and explored whether the effects are different for boys and girls. Specifically, we explored the effects on (1) combined exposure to violence in the school environment from school staff and peers, (2) severe physical violence from school staff towards students, injuries among students inflicted by school staff, emotional and sexual violence from school staff, and which specific acts of physical violence from school staff were impacted by the intervention, and (3) emotional, physical and sexual violence from peers towards students. We also examined reports of physical and emotional violence from school staff for evidence of replacement of one form of violence with another—that is, a reduction in staff use of physical violence with a simultaneous increase in staff use of emotional violence against students.

## Methods

The Good Schools Study (Devries et al. 2013) is registered at clinicaltrials.gov (NCT01678846) and was approved by the LSHTM Ethics Committee (6183) and the Uganda National Council for Science and Technology (SS2520). Further details about our methodology are published elsewhere (Devries et al. 2013, 2015).

### Study Setting and Timeline

Forty-two primary schools in Luwero District, Uganda, were included in the study. Cross-sectional surveys were conducted at schools in June/July 2012 and June/July 2014. The intervention was implemented over 18 months, between September/October 2012 and April/May 2014.

### Study Design and Participants

We carried out a two-arm cluster-randomised controlled trial with parallel assignment. Using the official 2010 list of all 268 primary schools in Luwero as our sampling frame, we excluded 105 schools with fewer than 40 registered primary 5 students and 20 schools with existing governance interventions. Forty-two schools were randomly selected and all agreed to participate in the study. Schools did not receive any inducement or incentive for participation (other than receiving the Toolkit intervention). Parents were informed that the study would be taking place and could opt their children out. Children themselves provided consent.

Current lists of all primary school grade 5, 6 and 7 students (aged about 11–14 years) were obtained from each school, and a simple random sample of up to 130 students from the lists was invited to participate. In schools with fewer than 130 P5–7 students, all students were invited for an interview. All those who could speak Luganda or English and who were deemed by interviewers to be able to understand the consent procedures were eligible. The flow of participants through the trial is described elsewhere (Devries et al. 2015).

### Interventions

The Good School Toolkit is a complex intervention which aims to foster change of operational culture at the school level, developed by the Ugandan NGO Raising Voices (Devries et al. 2015). The Toolkit is publicly available at www.raisingvoices.org. Briefly, the Toolkit consists of six steps designed to be implemented in sequence and draws on the Transtheoetical Model of behaviour change (Prochaska and Velicer 1997). The steps contain more than 60 different activities for staff, students and administration, focused around topics such as improving the school compound and creating a better learning environment, respect and understanding power relationships, improving teaching techniques, creating accountability, and learning non-violent methods of discipline. These are delivered by two staff and two student ‘protagonists’, who are chosen at the outset of the intervention to lead processes at each school. The protagonists receive ongoing support from Raising Voices staff. The intervention activities contain a number of established behaviour change techniques (Abraham and Michie 2008) such as setting goals, making action plans, rewards and reinforcement, and crucially, because it works with multiple actors within the school setting simultaneously, it creates social support for change. Schools not receiving the Toolkit were wait-listed to do so.

### Fidelity of Implementation

The 21 intervention schools completed the Good School Toolkit intervention during the 18-month implementation period. Appointed protagonists and head teachers from all schools participated in the Toolkit initiation training. Although the Toolkit six steps were completed in all schools, Raising Voices programme implementers reported that the quality and/or adoption of the steps did vary between schools. Raising Voices technical school support visits took place at the planned frequency in all schools, with an average of two support visits per school term of Toolkit implementation.

### Outcomes

Measures are derived from the International Society for the Prevention of Child Abuse and Neglect Child Abuse Screening Tool-Child Institutional (ICAST-CI) (International Society for the Prevention of Child Abuse and Neglect 2006) (reported in Table 1). The ICAST has been used extensively and validated in a variety of settings internationally. Measures were translated where necessary, and some items and time frames for recall added to capture the Ugandan context. All were pretested for understanding and piloted before the baseline survey (Devries et al. 2013).

**Table 1:** Measures

Variable name	Instrument, items	Coding
School staff violence		
Physical violence, severe physical violence* Time frame: past week, past school term, ever	Has a school staff member: hurt you or caused pain to you? Slapped you with a hand on your face or head as punishment? Slapped you with a hand on your arm or hand? Twisted your ear as punishment? Twisted your arm as punishment? Pulled your hair as punishment? Hit you by throwing an object at you? Hit you with a closed fist? Hit you with a stick? Caned you? Kicked you? Knocked you on the head as punishment? Made you dig, slash a field, or do other labour as punishment? Hit your fingers or hands with an object as punishment? Crushed your fingers or hands as punishment? Made you stand /kneel in a way that hurts to punish you? Made you stay outside for example in the heat or rain to punish you? Burnt you as punishment?* Taken your food away from you as punishment? Forced you to do something that was dangerous?* Choked you? Tied you up with a rope or belt at school?* Tried to cut you purposefully with a sharp object?* Severely beat you up?*	Coded 1 if answered yes to any of the items; 0 if answered no to all items.
Emotional violence Time frame: past week, past school term, ever	Has a school staff member: Cursed, insulted, shouted at or humiliated you? Referred to your skin colour/ gender/ religion/ tribe or health problems you have in a hurtful way? Stopped you from being with other children to make you feel bad or lonely*?* Tried to embarrass you because you were an orphan or without a parent? Embarrassed you because you were unable to buy things? Stole or broke or ruined your belongings? Threatened you with bad marks that you did not deserve? Accused you of witchcraft?	Coded 1 if answered yes to any of the items; 0 if answered no to all items.
Sexual violence Time frame: past week, past school term, ever	Has a school staff member: Teased you or made sexual comments about your breasts, genitals, buttocks or other body parts? Touched your body in a sexual way or in a way that made you uncomfortable? By ‘sexual way’ we mean touching you on your genitals, breasts or buttocks. Showed you pictures, magazines, or movies of people or children doing sexual things? Made you take your clothes off when it was not for a medical reason? Opened or took their own clothes off in front of you when they should not have done so? Kiss you when you did not want to be kissed? Make you touch their genitals, breasts or buttocks when you did not want to? Touch your genitals, breasts or buttocks when you did not want them to? Give you money/ things to do sexual things? Involve you in making sexual pictures or videos? Threaten or pressure you to have sex or do sexual things with them? Actually make you have sex with them by threatening or pressuring you, or by making you afraid of what they might do? Make you have sex with them by physically forcing you (have sex with you)?	Coded 1 if answered yes to any of the items; 0 if answered no to all items.
Any injury (moderate injury*, severe injury **) Time frame: past week, past school term, ever	You felt pain? You had bruising?* You had swelling?* You were bleeding?* You had cuts?* It was difficult to sit down on your buttocks?* It was difficult to walk?* You lost consciousness, even temporarily?** You suffered a dislocated, sprained, fractured or broken bone?** You had any other serious injury?** You had to get medical attention, for example from the health worker or hospital? ** You had to stay home from school?	Coded 1 if answered yes to any of the items; 0 if answered no to all items.
Peer violence		
Emotional violence/neglect Time frame: past week, past school term, ever	Has anyone besides a school staff member: Insulted you, or called you rude or hurtful names? Accused you of witchcraft? Locked you out or made you stay outside? Not given you food? Perpetrator asked after each act; multiple perpetrators could be mentioned.	Coded 1 if answered yes to any of the items; 0 if answered no to all items.
Physical violence Time frame: past week, past school term, ever	Has anyone besides a school staff member: Twisted your arm or any other body part, slapped you, pushed you or thrown something at you? Punched you, kicked you, or hit you with a closed fist? Hit you with an object, such as a stick or a cane, or whipped you? Cut you with a sharp object or burnt you? Perpetrator asked after each act; multiple perpetrators could be mentioned.	Coded 1 if answered yes to any of the items; 0 if answered no to all items.
Sexual violence Time frame: past week, past school term, ever	Has anyone besides a school staff member: Disturbed or bothered you by making sexual comments about you? Kissed you, when you did not want them to? Touched your genitals or breasts when you did not want them to, or in a way that made you uncomfortable? Threaten or pressure you to make you do something sexual with them? Make you have sex with them, because they threatened or pressured you? Had sex with you, by physically forcing you? Perpetrator asked after each act; multiple perpetrators could be mentioned.	Coded 1 if answered yes to any of the items; 0 if answered no to all items.

### Procedures

Interviewers collected data from students, during baseline and endline cross-sectional surveys, in one-on-one interviews conducted within sight but out of earshot of others at the school. As literacy levels were low in schools, questionnaires were programmed into mobile phones or tablet computers and read aloud to students; interviewers entered responses. Students could complete the interview in either Luganda or English. The survey took about 45 min. Students were advised that they could stop the interview at any time, and interviewers were trained to stop or take a break if a student became distressed during the interview. All children were offered counselling regardless of what they disclosed.

### Child Protection Procedures

In this study, potential risks related to the intervention itself were minimal, but during survey data collection, we detected children in need of support from child protective services because they had experienced abuse. Children were informed during the consent process that their details might be passed on to child protection officers. Referrals were based on predefined criteria agreed with service providers, related to the severity and timing of violence reported (Child et al. 2014; Devries et al. 2015.

### Sample Size Calculations

Allowing for a loss to follow-up of two schools per arm, and conservatively assuming interviews with 60 students per school, with a prevalence of past week physical violence of 50% and an intracluster correlation coefficient of 0.06 (from our baseline survey) (Devries et al. 2014a), we had 80% power to detect a 13% difference in the prevalence of reported violence between the intervention and control arms with 5% statistical significance.

### Randomisation and Masking

Stratified block randomisation was done (Devries et al. 2015). Due to the nature of the intervention, it was not possible to mask participants. Given the nature of the intervention, interviewers should also be considered unmasked.

## Statistical Analysis

We performed an intention to treat analysis using data from our cross-sectional follow-up survey. All analyses were performed in Intercooled Stata 13.1 (Stata Corp 2014). Data were collected using a survey programmed into tablet computers with algorithms designed to eliminate erroneous skips. Analysis was performed using individual-level student data, accounting for clustering of students within schools using mixed effects regression models. Adjusted analyses are presented and control for baseline school-level means of the violence outcome (modelled as a continuous variable), whether or not students had a disability, their school’s location (urban or rural). To explore possible sex differences in effects of the Toolkit on violence outcomes, interaction terms were fitted (sex with study arm), and adjusted models with and without interaction terms were compared using Likelihood ratio tests. Stratum specific odds ratios for boys and girls are calculated based on models with interaction terms.

### Demographic Characteristics of Students at Baseline

Student characteristics were evenly distributed across study arms at baseline (Table 2). At baseline, students were 13 years old, on average, about 7% had some form of disability (most commonly difficulties with sight, followed by hearing), and less than half had eaten three meals on the previous day. About 19% of boys reported working more than 2 h a day outside of school, and about 15% of girls reported the same. Most students walked to school with someone they know.

**Table 2:** Demographic characteristics at baseline

	Boys		Girls	
	Control (*n* = 881) *n* (%)	Intervention (*n* = 885) *n* (%)	Control (*n* = 1018) *n* (%)	Intervention (*n* = 1036) *n* (%)
Demographics				
Age (years), mean (SD)	13.1 (1.5)	13.3 (1.5)	12.8 (1.4)	12.8 (1.4)
School class				
5	360 (41.3)	383 (42.7)	343 (34.0)	356 (38.4)
6	287 (32.9)	287 (32.0)	410 (40.6)	357 (38.5)
7	225 (25.8)	227 (25.3)	257 (25.5)	214 (23.1)
Disability	71 (8.1)	68 (7.6)	71 (7.0)	61 (6.6)
Some disability				
Meals eaten previous day				
1 meal	105 (12.0)	125 (13.9)	145 (14.4)	141 (15.2)
2 meals	383 (43.9)	381 (42.5)	360 (35.7)	319 (34.4)
3+ meals	384 (44.0)	391 (43.6)	504 (50.0)	467 (50.4)
Hours of work each day				
Less than 1 h	337 (38.7)	314 (35.1)	451 (45.0)	321 (34.8)
1–2 h	362 (41.6)	412 (46.0)	418 (41.7)	456 (49.5)
More than 2 h	171 (19.7)	169(18.9)	133 (13.3)	145 (15.7)
Mode of transport to school				
Other	44 (5.2)	52 (6.0)	18 (1.8)	25 (2.7)
Walking alone	201 (23.5)	242 (28.0)	239 (24.3)	208 (22.6)
Walking with someone you know	528 (61.8)	539 (62.4)	633 (64.4)	614 (66.7)
Board at school	82 (9.6)	31 (3.6)	93 (9.5)	73 (7.9)
Absence from school in previous week				
1 or more days missed	168 (19.6)	228 (25.6)	179 (18.4)	197 (21.5)
Outcomes (at baseline)				
Total school violence, past week	522 (59.9)	534 (59.5)	656 (65.0)	551 (59.4)
Any violence from school staff, past week	468 (53.7)	480 (53.5)	600 (59.4)	509 (54.9)
Physical violence from school staff, past week	448 (51.4)	470 (52.4)	580 (57.4)	492 (53.1)
Emotional violence from school staff, past week	98 (11.2)	83 (9.3)	138 (13.7)	96 (10.4)
Sexual violence from school staff, past week	6 (0.7)	0 (0)	11 (1.1)	4 (0.4)
Any injury from school staff, past week	194 (24.2)	232 (27.4)	280 (29.6)	230 (26.1)
Moderate injury from school staff, past week	38 (4.7)	46 (5.4)	74 (7.8)	65 (7.4)
Severe injury from school staff, past week	10 (1.3)	9 (1.1)	10 (1.1)	9 (1.0)
Any violence from peers, past week	198 (22.7)	174 (19.4)	235 (23.3)	175 (18.9)
Physical violence from peers, past week	71 (8.1)	66 (7.4)	109 (10.8)	72 (7.8)
Emotional violence from peers, past week	162 (18.6)	141 (15.7)	168 (16.6)	129 (13.9)
Sexual violence from peers, past week	8 (0.9)	2 (0.2)	12 (1.2)	12 (1.3)

### Baseline Levels of Violence

The prevalence of violence in school was very high, with 60% or more students reporting some form of violence in the past week (Table 2). Past week staff physical violence was far more commonly reported than peer physical violence in this sample; for emotional violence, both boys and girls reported higher levels of peer perpetration. Between 25 and 30% of boys and girls reported some form of injury from school staff in the past week, with girls slightly more commonly reporting moderate injuries. Sexual violence was less commonly reported relative to other forms of violence, although case numbers are still very high considering the past week time frame of reporting. For boys, outcome variables were balanced across arms, but for girls, there was some suggestion that violence outcomes were more common in the control versus intervention arms.

## Results

**Fig. 1:**
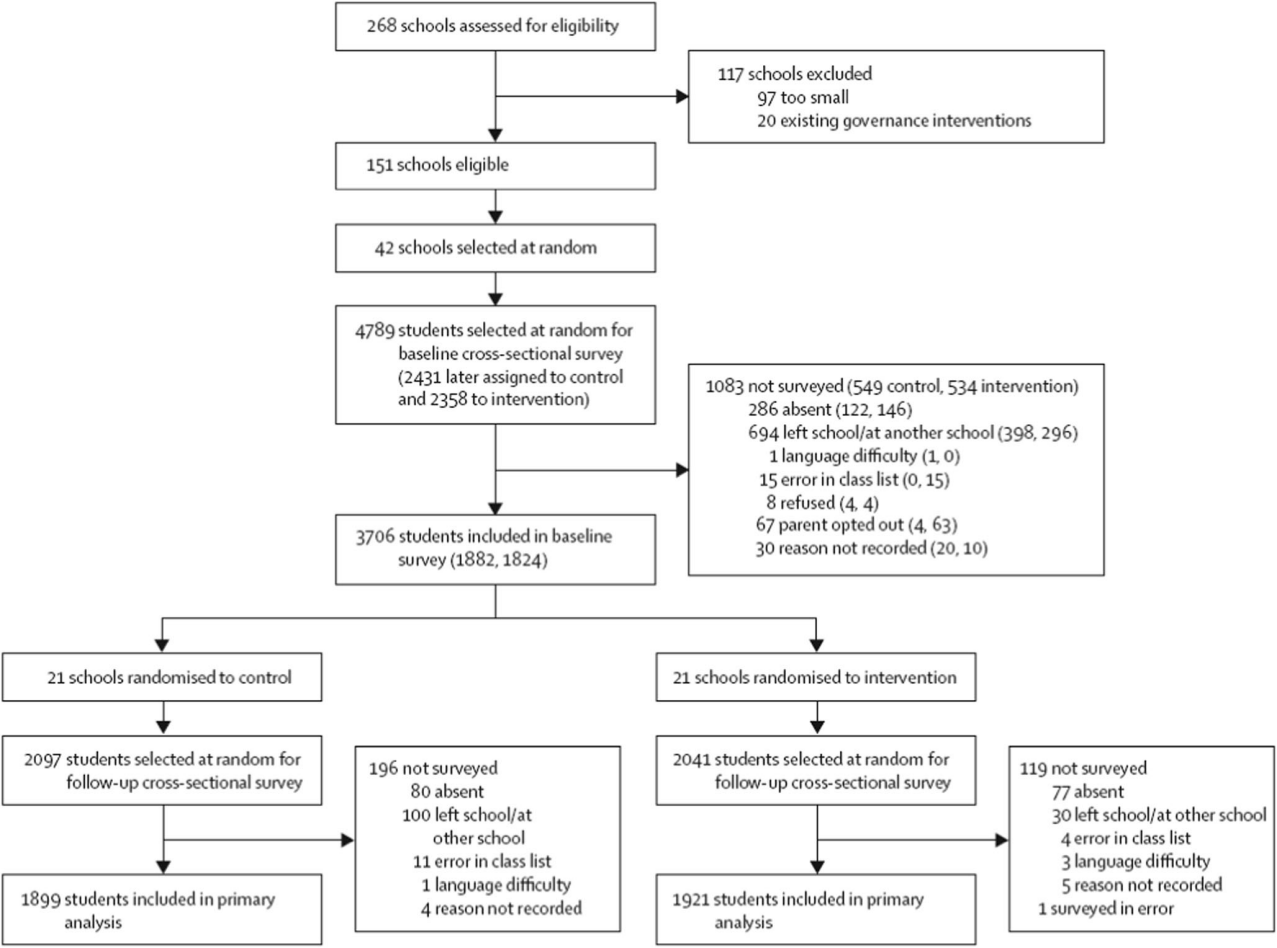
Flow of participants through the trial

Forty-two schools participated in the baseline and endline survey, and 92.3% (*n* = 3820) of sampled students were interviewed at endline. The flow of participants through the trial is described in Fig. 1. The quantity of missing data was low (<3%) and similar across study groups for all measures presented.

### Intervention Effects on Combined Violence from Staff and Peers in the School Environment

The intervention was associated with a reduction in overall levels of ‘any’ form of violence (physical, emotional, sexual combined) from school staff and/or peers, over both the past week and past term time frames (Table 3). The magnitude of the reduction was larger in boys than girls over both time frames, with strong evidence of a statistically significant interaction effect by sex. Considering any form of violence from school staff, there was a reduction in intervention schools for both boys and girls over the past week and past term time frames; again, the magnitude of the reduction was larger for boys than for girls. Students in the Toolkit intervention schools displayed significantly lower peer violence over the past week and past term, but there was no evidence of a differential effect for boys relative to girls.

**Table 3:** Effect of the intervention on summary violence outcomes

	Total				Boys			Girls			
Outcome	Control (*n* = 1899) *n* (%)	Intervention (*n* = 1921) *n* (%)	aOR^a^ (95%CI)	*p*	Control (*n* = 881) *n* (%)	Intervention (*n* = 885) *n* (%)	aOR^b^ (95%CI)	Control (*n* = 1018) *n* (%)	Intervention (*n* = 1036) *n* (%)	aOR^b^ (95%CI)	LR test *p*
Any violence, staff or peers, past week	1077 (56.7)	773 (40.2)	0.44 (0.29–0.66)	<0.0001	507 (57.6)	317 (35.8)	0.34 (0.22–0.53)	570 (56.0)	456 (44.0)	0.55 (0.36–0.84)	<0.0001
Any violence, staff or peers, past term	1616 (85.1)	1294 (67.4)	0.31 (0.17–0.55)	<0.0001	766 (87.0)	553 (62.5)	0.20 (0.11–0.37)	850 (83.5)	741 (71.5)	0.44 (0.24–0.82)	<0.0001
Any staff violence, past week	953 (50.2)	634 (33.0)	0.41 (0.26–0.64)	<0.0001	441 (50.1)	258 (29.2)	0.34 (0.21–0.54)	512 (50.3)	376 (36.3)	0.49 (0.31–0.77)	0.009
Any violence, staff, past term	1543 (81.3)	1185 (61.7)	0.31 (0.18–0.54)	<0.0001	728 (82.6)	489 (55.3)	0.21 (0.12–0.37)	815 (80.1)	696 (67.2)	0.45 (0.26–0.80)	<0.0001
Any peer violence, past week	435 (22.9)	337 (17.5)	0.70 (0.51–0.96)	0.026	210 (23.8)	148 (16.7)	0.62 (0.43–0.89)	225 (22.1)	189 (18.2)	0.77 (0.54–1.09)	0.192
Any peer violence, past term	677 (35.7)	558 (29.1)	0.68 (0.49–0.94)	0.019	345 (39.2)	269 (30.4)	0.61 (0.43–0.88)	332 (32.6)	289 (27.9)	0.74 (0.52–1.05)	0.184

^a^Adjusted for sex, baseline level of school violence, past-term ones adjusted for past week baseline school violence, disability and urban/rural school

^b^Adjusted for all except sex

### Intervention Effects on Violence from Staff Towards Students

In general, fewer students in the intervention arm relative to the control arm reported emotional violence; this association was not statistically significant over the past week, but was significant over the past term (Table 4). There was no evidence of a differential effect in boys and girls. The most commonly reported specific acts of emotional violence were being cursed, insulted, shouted at or humiliated.

**Table 4:** Emotional violence from school staff to students, reported by students

	Total				Boys			Girls			
Act	Control (*n* = 1899) *n* (%)	Intervention (*n* = 1921) *n* (%)	aOR^a^ (95%CI)	*p*	Control (*n* = 881) *n* (%)	Intervention (*n* = 885) *n* (%)	aOR^b^ (95%CI)	Control (*n* = 1018) *n* (%)	Intervention (*n* = 1036) *n* (%)	aOR^b^ (95%CI)	LR test *p*
Any past week emotional violence	179 (9.4)	140 (7.3)	0.78 (0.49–1.21)	0.272	78 (8.9)	55 (6.2)	0.68 (0.40–1.16)	101 (9.9)	85 (8.2)	0.85 (0.52–1.40)	0.354
Any past term emotional violence	347 (18.3)	260 (13.5)	0.68 (0.47–0.99)	0.044	164 (18.6)	113 (12.8)	0.60 (0.40–9.92)	183 (18.0)	147 (14.2)	0.76 (0.50–1.14)	0.218
Specific items, past week:											
Cursed, insulted, shouted at or humiliated you?	109 (5.7)	78 (4.1)	0.69 (0.45–1.07)	0.098	54 (6.1)	31 (3.5)	0.54 (0.31–0.94)	55 (5.4)	47 (4.5)	0.84 (0.50–1.41)	0.151
Referred to your skin colour/gender/religion/tribe or health problems you have in a hurtful way?	33 (1.7)	25 (1.3)	0.69 (0.34–1.38)	0.294	11 (1.3)	8 (0.9)	0.66 (0.24–1.85)	22(2.2)	17 (1.6)	0.70 (0.32–1.55)	0.923
Stopped you from being with other children to make you feel bad or lonely*?*	7 (0.4)	16 (0.8)	2.79 (0.95–8.20)	0.062	4 (0.5)	7 (0.8)	2.06 (0.52–8.15)	3 (0.3)	9 (0.9)	3.79 (0.89–16.12)	0.506
Tried to embarrass you because you were an orphan or without a parent?	2 (0.1)	1(0.05)			0	1 (0.1)		2 (0.2)	0		
Embarrassed you because you were unable to buy things?	13 (0.7)	15 (0.8)	1.14 (0.49–2.69)	0.758	5 (0.6)	4 (0.5)		8 (0.8)	11 (1.1)	1.39 (0.51–3.82)	0.520
Stole or broke or ruined your belongings?	36 (1.9)	21 (1.1)	0.80 (0.23–2.77)	0.721	8 (0.9)	8 (0.9)		28 (2.8)	13 (1.3)	0.66 (0.18–2.41)	0.302
Threatened you with bad marks that you did not deserve?	12 (0.6)	10 (0.5)	0.79 (0.23–2.71)	0.707	5 (0.6)	5 (0.6)	0.94 (0.20–4.36)	7 (0.7)	5 (0.5)	0.70 (0.16–2.95)	0.710
Accused you of witchcraft?	3 (0.2)	3 (0.2)			1 (0.1)	0		2 (0.2)	3 (0.3)		

^a^Controlled for school-level baseline mean value of variable, school, sex, rural/urban, disability and baseline violence prevalence

^b^Adjusted for all except sex. No OR displayed for less than 20 cases

Students in the intervention arm reported less physical violence from school staff over the past week and past term (Table 5). Statistically significant reductions were reported by both boys and girls, but the magnitude of the reduction was larger in boys than girls. This was mainly driven by a reduction in caning, which was the most common act of physical violence from school staff reported by both boys and girls in the past week. There were also significant past week reductions in being slapped on the face or head, and being hit by having an object thrown at you.

**Table 5:** Physical violence from school staff to students, reported by students

	Total				Boys			Girls			
Act	Control (*n* = 1899) *n* (%)	Intervention (*n* = 1921) *n* (%)	aOR^a^ (95%CI)	*p*	Control (*n* = 881) *n* (%)	Intervention (*n* = 885) *n* (%)	aOR^b^ (95%CI)	Control (*n* = 1018) *n* (%)	Intervention (*n* = 1036) *n* (%)	aOR^b^ (95%CI)	LR test *p*
Any physical violence, past week	924 (48.7)	595 (31.0)	0.39 (0.25–0.62)	<0.0001	422 (47.9)	242 (27.3)	0.33 (0.21–0.54)	502 (49.3)	353 (34.1)	0.45 (0.28–0.72)	0.033
Any physical violence, past term	1528 (80.5)	1157 (60.2)	0.31 (0.18–0.53)	<0.0001	721 (81.8)	475 (53.7)	0.20 (0.11–0.36)	807 (79.3)	682 (65.8)	0.44 (0.25–0.78)	<0.0001
Any severe physical violence, past week	31 (1.63)	13 (0.68)	0.40 (0.15–1.05)	0.063	6 (0.7)	5 (0.6)	0.77 (0.19–3.13)	25 (2.5)	8 (0.8)	0.31 (0.11–0.91)	0.227
Any severe physical violence, past term	58 (3.1)	31 (1.6)	0.54 (0.29–1.00)	0.049	29 (3.3)	11 (1.2)	0.38 (0.16–0.86)	29 (2.9)	20 (1.9)	0.70 (0.34–1.45)	0.174
Specific items, past week:											
Hurt you or caused pain to you?	70 (3.7)	65 (3.4)	0.79 (0.30–2.03)	0.620	22 (2.5)	25 (2.8)	0.94 (0.32–2.72)	48 (4.7)	40 (3.9)	0.71 (0.27–1.91)	0.468
Slapped you with a hand on your face or head as punishment?	83 (4.4)	48 (2.5)	0.40 (0.21–0.77)	0.006	46 (5.2)	19 (2.2)	0.40 (0.21–0.77)	37 (3.6)	29 (2.8)	0.78 (0.42–1.44)	0.077
Slapped you with a hand on your arm or hand?	35 (1.8)	27 (1.4)	0.61 (0.31–1.22)	0.159	18 (2.0)	15 (1.7)	0.64 (0.28–1.48)	17 (1.7)	12 (1.2)	0.58 (0.24–1.39)	0.844
Twisted your ear as punishment?	79 (4.2)	88 (4.6)	0.95 (0.57–1.58)	0.832	42 (4.8)	36 (4.1)	0.70 (0.38–1.30)	37 (3.6)	52 (5.0)	1.23 (0.68–2.24)	0.080
Twisted your arm as punishment?	9 (0.5)	2 (0.1)			3 (0.3)	1 (0.1)		6 (0.6)	1 (0.1)		
Pulled your hair as punishment?	20 (1.1)	14 (0.7)	0.70 (0.33–1.46)	0.342	7 (0.8)	11 (1.2)	1.57 (0.58–4.24)	13 (1.3)	3 (0.3)	0.23 (0.06–0.85)	0.011
Hit you by throwing an object at you?	37 (2.0)	17 (0.9)	0.43 (0.23–0.77)	0.005	14 (1.6)	3 (0.3)	0.19 (0.06–0.69)	23 (2.3)	14 (1.4)	0.57 (0.29–1.13)	0.117
Hit you with a closed fist?	15 (0.8)	14 (0.7)	0.89 (0.37–2.2)	0.803	4 (0.5)	7 (0.8)	1.68 (0.44–6.35	11 (1.1)	7 (0.7)	0.61 (0.21–1.78)	0.197
Hit you with a stick?	142 (7.5)	79 (4.1)	0.43 (0.18–1.04)	0.062	49 (5.6)	13 (1.5)	0.22 (0.08–0.61)	93 (9.1)	66 (6.4)	0.55 (0.22–1.35)	0.008
Caned you?	774 (40.8)	433 (22.5)	0.36 (0.22–0.57)	<0.0001	350 (39.7)	172 (19.4)	0.30 (0.19–0.50)	424 (41.7)	261 (25.2)	0.41 (0.25–0.66)	0.062
Kicked you?	9 (0.5)	4(0.2)			5 (0.6)	0		4 (0.4)	4 (0.4)		
Knocked you on the head as punishment?	52 (2.7)	66 (3.4)	1.01 (0.55–1.87)	0.977	25 (2.8)	29 (3.3)	0.91 (0.44–1.90)	27 (2.7)	37 (3.6)	1.10 (0.54–2.23)	0.634
Made you dig, slash a field, or do other labour as punishment?	98 (5.2)	91(4.7)	0.97 (0.55–1.71)	0.910	45 (5.1)	34 (3.8)	0.77 (0.39–1.50)	53 (5.2)	57 (5.5)	1.15 (0.61–2.15)	0.196
Hit your fingers or hands with an object as punishment?	30 (1.6)	26 (1.4)	0.80 (0.41–1.56)	0.507	13 (1.5)	10 (1.1)	0.70 (0.28–1.78)	17 (1.7)	16 (1.5)	0.87 (0.39–1.95)	0.706
Crushed your fingers or hands as punishment?	1 (0.05)	2 (0.1)			1 (0.1)	1 (0.1)		0	1 (0.1)		
Made you stand /kneel in a way that hurts to punish you?	36 (1.9)	33 (1.7)	0.82 (0.45–1.52)	0.535	15 (1.7)	19 (2.2)	1.13 (0.52–2.47)	21 (2.1)	14 (1.4)	0.61 (0.28–1.32)	0.208
Made you stay outside for example in the heat or rain to punish you?	18 (1.0)	13 (0.7)	0.58 (0.17–2.01)	0.391	6 (0.7)	3 (0.3)		12 (1.2)	10 (1.0)	0.68 (0.19–2.43)	0.583
Burnt you as punishment?^a^	0	0			0	0		0	0		
Taken your food away from you as punishment?	1 (0.05)	0			0	0		1 (0.1)	0		
Forced you to do something that was dangerous?^a^	0	1 (0.05)			0	1 (0.1)		0	0		
Choked you?^a^	0	1 (0.05)			0	1(0.1)		0	0		
Tied you up with a rope or belt at school?	0	0			0	0		0	0		
Tried to cut you purposefully with a sharp object?^a^	0	0			0	0		0	0		
Severely beat you up?^a^	31 (1.63)	11 (0.6)	0.35 (0.13–0.96)	0.042	6 (0.7)	3 (0.3)	0.48 (0.10–2.38)	25 (2.5)	8 (0.8)	0.32 (0.11–0.94)	0.611

^a^Controlled for school-level baseline mean value of variable, school, sex, rural/urban, disability and baseline violence prevalence

^b^Adjusted for all except sex. No OR displayed for less than 20 cases

There was a borderline significant reduction in severe physical violence from school staff in the past week, which reached statistical significance over the past term time frame. This was mainly driven by a reduction in being ‘severely beaten up’. There was no evidence of a differential effect between girls and boys, but case numbers were relatively low. In terms of sexual violence, the number of reported cases where school staff were the perpetrators over the past week and past term was few in number, and no clear pattern of effect emerged (Table 6).

**Table 6:** Sexual violence from school staff to students, reported by students

	Total				Boys			Girls			
Act	Control (*n* = 1899) *n* (%)	Intervention (*n* = 1921) *n* (%)	aOR^a^ (95%CI)	*p*	Control (*n* = 881) *n* (%)	Intervention (*n* = 885) *n* (%)	aOR^b^ (95%CI)	Control (*n* = 1018) *n* (%)	Intervention (*n* = 1036) *n* (%)	aOR^b^ (95%CI)	LR test *p*
Any sexual violence, past week	13 (0.7)	3 (0.2)			4 (0.5)	0		9 (0.9)	3 (0.3)		
Any sexual violence past term	16 (0.8)	16 (0.8)	1.04 (0.48–2.25)	0.918	7 (0.8)	6 (0.7)	0.85 (0.27–2.70)	9 (0.9)	10 (1.0)	1.20 (0.46–3.10)	0.648

^a^Controlled for school-level baseline mean value of variable, school, sex, rural/urban, disability and baseline violence prevalence

^b^Adjusted for all except sex. No OR displayed for less than 20 cases

### Intervention Effects on Injuries Among Students from School Staff

Students in the intervention arm reported significantly fewer injuries from school staff in the past week and past term (Table 7). The magnitude of the reduction is higher in boys than girls over the past term time frame (significant interaction effect). Moderate injuries were also lower in Toolkit intervention school students over the past week and past term, again with evidence of slightly higher effects in boys than girls. There was no evidence of a reduction in severe injuries in the intervention versus control arm over the past term timeframe (and too few cases to estimate effects over the past week time frame). Reductions in injury were driven mainly by reduced reporting of pain and swelling.

**Table 7:** Injuries inflicted by school staff on students, reported by students who experienced physical or sexual violence from school staff (*n* = 3217)

	Total				Boys			Girls			
Act	Control (*n* = 1729) *n* (%)	Intervention (*n* = 1488) *n* (%)	aOR^a^ (95%CI)	*p*	Control (*n* = 814) *n* (%)	Intervention (*n* = 687) *n* (%)	aOR^b^ (95%CI)	Control (*n* = 915) *n* (%)	Intervention (*n* = 801) *n* (%)	aOR^b^ (95%CI)	LR test *p*
Any injury, past week (*n* = 3217)	490 (28.3)	247 (16.6)	0.44 (0.26–0.76)	0.003	217 (26.7)	91 (13.3)	0.36 (0.20–0.65)	273 (29.8)	156 (19.5)	0.51 (0.29–0.90)	0.073
Severe injury, past week (*n* = 3217)	5 (0.3)	3 (0.2)			5 (0.6)	0		2 (0.2)	1 (0.1)		
Moderate injury past week (*n* = 3217)	105 (6.1)	46 (0.1)	0.48 (0.28–0.84)	0.010	46 (5.7)	14 (2.0)	0.32 (0.15–0.69)	59 (6.5)	32 (4.0)	0.61 (0.33–1.13)	0.099
Any injury, past term (*n* = 3217)	1161 (67.2)	937 (63.0)	0.52 (0.34–0.80)	0.003	550 (67.6)	406 (59.1)	0.34 (0.21–0.55)	611 (66.8)	531 (66.3)	0.74 (0.47–1.17)	<0.0001
Severe injury, past term (*n* = 3217)	66 (3.8)	49 (3.3)	1.17 (0.66–2.08)	0.594	29 (3.6)	18 (2.6)	1.01 (0.38–2.70)	37 (4.0)	31 (3.9)	1.25 (0.63–2.49)	0.728
Moderate injury past term (*n* = 3217)	397 (23.0)	276 (18.6)	0.69 (0.50–0.95)	0.022	181 (22.2)	103 (15.0)	0.45 (0.30–0.70)	216 (23.6)	173 (21.6)	0.90 (0.62–1.29)	0.003
Specific items, past week:											
You felt pain?	477 (27.8)	234 (15.7)	0.43 (0.24–0.73)	0.002	213 (26.2)	87 (12.7)	0.35 (0.20–0.63)	264 (28.9)	147 (18.4)	0.50 (0.28–0.87)	0.071
You had bruising?^c^	–				2 (0.2)	1 (0.1)		11 (1.1)	6 (0.6)		
You had swelling?^c^	70 (4.1)	30 (2.0)	0.49 (0.25–0.96)	0.039	31 (3.8)	8 (1.2)	0.29 (0.11–0.76)	39 (4.3)	22 (2.8)	0.64 (0.30–1.36)	0.102
You were bleeding?^c^	5 (0.3)	1 (0.05)			3 (0.3)	0		2 (0.2)	1 (0.1)		
You had cuts?^c^	2 (0.1)	0			1 (0.1)	0		1	0		
It was difficult to sit down on your buttocks?^c^	49 (2.8)	24 (1.6)	0.55 (0.27–1.10)	0.091	19 (2.3)	6 (0.9)	0.33 (0.11–0.95)	30 (3.3)	18 (2.3)	0.69 (0.31–1.50)	0.180
It was difficult to walk?^c^	3 (0.2)	7 (0.4)			3 (0.3)	0		3 (0.3)	4 (0.4)		
You lost consciousness, even temporarily? ^d^	0	0			0	0		0	0		
You suffered a dislocated, sprained, fractured or broken bone? ^d^	1 (0.05)	0			0	0		1 (0.1)	0		
You had any other serious injury?^d^	0	0			0	0		0	0		
You had to get medical attention, for example from the health worker or hospital?^d^	6 (0.3)	1 (0.05)			5 (0.6)	0		1 (0.1)	1(0.1)		
You had to stay home from school?	9 (0.5)	4 (0.2)			7 (0.8)	0		2 (0.2)	4 (0.4)		

^a^Controlled for school-level baseline mean value of variable, school, sex, rural/urban, disability and baseline violence prevalence

^b^Adjusted for all except sex. No OR displayed for less than 20 cases

^c^Moderate injury

^d^Severe injury

### Intervention Effects on Peer Violence

Student reports of emotional violence from peers over the past week and past term were lower in the intervention arm, with no evidence of any difference in effect in boys and girls (Table 8). Peer physical violence over the past week was not statistically significantly different between control and intervention arms; however, the association approached borderline statistical significance over the past term time frame. There was also some suggestion of a potential interaction over the past term time frame, which did not reach statistical significance but where effects were more pronounced in boys than girls. Case numbers of peer sexual violence over the past week and past term were low, but results suggest that the intervention was associated with a borderline increase in reports of sexual violence for girls in particular, over both time frames, although this did not reach statistical significance.

**Table 8:** Peer violence

	Total				Boys			Girls			
Act	Control (*n* = 1899) *n* (%)	Intervention (*n* = 1921) *n* (%)	aOR^a^ (95%CI)	*p*	Control (*n* = 885) *n* (%)	Intervention (*n* = 881) *n* (%)	aOR^b^ (95%CI)	Control (*n* = 1036) *n* (%)	Intervention (*n* = 1018) *n* (%)	aOR^b^ (95%CI)	LR test *p*
Any emotional peer violence, past week	322 (17.0)	241 (12.6)	0.68 (0.51–0.90)	0.007	170 (19.3)	115 (13.0)	0.59 (0.42–0.83)	152 (14.9)	126 (12.2)	0.77 (0.55–1.08)	0.731
Any peer physical violence, past week	182 (9.6)	145 (7.6)	0.77 (0.53–1.12)	0.169	72 (8.2)	60 (6.8)	0.82 (0.51–1.30)	110 (10.8)	85 (8.2)	0.74 (0.48–1.12)	0.669
Any peer sexual violence, past week	9 (0.5)	12 (0.6)	1.25 (0.52–2.99)	0.616	5 (0.6)	2 (0.2)	0.38 (0.07–1.96)	4 (0.4)	10 (1.0)	2.33 (0.73–7.51)	0.061
Any emotional peer violence, past term	498 (26.2)	401 (20.9)	0.67 (0.49–0.92)	0.012	257 (29.2)	207 (23.4)	0.67 (0.48–0.95)	241 (23.7)	194 (18.7)	0.68 (0.48–0.95)	0.964
Any peer physical violence, past term	321 (16.9)	270 (14.1)	0.75 (0.55–1.02)	0.067	165 (18.7)	121 (13.7)	0.63 (0.44–0.90)	156 (15.3)	149 (14.4)	0.88 (0.62–1.26)	0.064
Any peer sexual violence, past term	10 (0.5)	19 (1.0)	2.01 (0.92–4.41)	0.080	5 (0.6)	3 (0.3)	0.64 (0.15–2.72)	5 (0.5)	16 (1.5)	3.39 (1.22–9.39)	0.056

^a^Controlled for school-level baseline mean value of variable, school, sex, rural/urban, disability and baseline violence

^b^Adjusted for all except sex. No OR displayed for less than 20 cases

## Discussion

Children who were in schools that used the Toolkit experienced reduced odds of a range of different forms of violence in the school environment—severe physical violence and injury from school staff, emotional violence from school staff, emotional violence from peers and probably for boys, physical violence from peers. Our exploratory analyses suggest that some of the effects on various forms of violence of the Toolkit are larger in boys versus girls. For sexual violence, there was some suggestion that the use of the Toolkit was associated with an increase in reports of cases of peer sexual violence among girls, but case numbers are low and this finding should be interpreted with caution.

The Toolkit represents one of the first interventions that has shown promise to successfully reduce physical and emotional violence from both school staff and peers in the school environment. The Toolkit is designed to be a holistic, comprehensive intervention which changes school culture. Our results are consistent with the limited number of studies which have tested whole school interventions to reduce students’ aggressive behaviour and improving social interaction outcomes, reviewed by Bonnell et al. (Bonell et al. 2013). However, reviewed studies are from high-income contexts, and none of the interventions included in this review examined effects separately by sex, so it is unknown to what extent they were effectively reaching both boys and girls (Bonell et al. 2013). Ttofi and Farrington also reviewed school-based anti-bullying programs and found that more intensive programs, programs which included parent meetings, firm disciplinary methods and improved supervision of children, were more effective in reducing bullying versus those without and programs working with peers were actually harmful (Ttofi and Farrington 2011). Sex differences were also not examined in this review, so any differential effects for boys and girls are unclear (Ttofi and Farrington 2011).

The suggestion of an increase in sexual violence reports by girls in intervention schools could have several interpretations and is an area where further exploration is required. A worrying but unlikely possibility is that the reduction in harsh punishments from staff towards boys has removed a deterrent preventing sexual aggression from boys towards female students. However, if harsh punishment from staff was acting as a deterrent for peer violence which has now been removed as a result of the intervention, then we might reasonably expect an increase in all forms of peer violence. Instead, the data show reductions in peer violence. A more plausible explanation for the increase in girls’ reports of sexual violence is that the intervention has created an environment where they feel more able to disclose their experiences. The Toolkit is associated with increased feelings of safety and well-being in school (Devries et al. 2015), and it does contain activities which support schools to improve their internal systems to deal with disclosures.

It remains unclear why there may be sex differences in the effects of the Toolkit—the Toolkit explicitly encourages equal participation of girls and boys in student activities, although there is no separate or dedicated module on encouraging gender equality. The lesser effect in girls may reflect the degree to which they participate in the intervention or could also reflect competing pressures and other experiences outside the school—in this context, prevailing gender norms dictate that girls bear responsibility for household duties and caring for younger siblings (Evans 2012), which may take up a large amount of time, possibly increasing absenteeism and tiredness and concentration difficulties. This may limit the ability of girls to effectively participate in intervention activities. However, boys are also often responsible for household tasks such as fetching water and carrying wood (Evans 2012), so this potential link needs further investigation. Girls also may be more exposed to other forms of violence outside the school environment, which might interact with their exposure to violence inside school (Devries et al. 2014a).

Corporal punishment takes place in schools for a variety of reasons, including as discipline in response to fighting between peers, non-compliance or misbehaviour in class, lateness, poor grades, lack of attention or poor performance in class (Breen et al. 2015; Malak et al. 2015). Some of these ‘reasons’, for example, violence between peers, seem to have been reduced more in boys. This may have led to a knock-on effect of also further reducing staff violence against boys. If girls, on the other hand, are punished more often for inattention or lateness, these might be less amenable to a school-based intervention and more influenced by the social and economic realities faced by families in this context. Further investigation is needed.

### Strengths and Limitations

Our study has a number of strengths, and of course, some limitations. This analysis is exploratory, and we have limited power to detect sex differences in the effects of the Toolkit. The specific acts of violence reported are single-item measures and are therefore subject to limitations associated with use of single-item measures, such as unknown biases in interpretation and random measurement error. We have also conducted a large number of statistical tests, thus increasing the likelihood that some findings will be due to chance. Therefore, findings should be considered in terms of consistency of trends and plausibility of results. We used valid, reliable instruments to measure exposure to violence, which are widely used internationally; we also pretested and piloted measures prior to use. Similar to other studies on violence, all of these measures are self-report. We intentionally chose student reports of experience of violence, rather than staff reports of perpetration, as the more conservative measure of intervention effect. We trained interviewers extensively in non-judgemental data collection techniques; however, it is still likely that more stigmatised forms of violence, such as sexual violence, are under-reported. This represents some of the first rigorous data collected on this topic in Uganda and in the region, and adds to our understanding of how the only rigorously evaluated intervention to reduce violence from school staff towards students is working, and where it could be further strengthened.

### Implications and Conclusion

Despite the encouraging overall effects in both boys and girls, the overall levels of violence experienced by these primary school students remain extremely high. Further work is urgently needed to investigate ways to further reduce this violence and to augment this intervention so that it is even more effective. The Toolkit is a promising intervention to reduce a range of forms of violence against children in schools and is seen to be effective for both boys and girls. Additional research is needed to understand why it is more effective in boys versus girls and to understand how it could be strengthened to further reduce school violence against girls in particular.

## Abbreviations

aOR: Adjusted odds ratio
ICAST-CI: International Society for the Prevention of Child Abuse and Neglect Child Abuse Screening Tool-Child Institutional
P: Primary
NGO: Non-Governmental Organisation
CHAI: Child Health Advocacy International
